# Appraising Pulmonary Function and Hand Grip Strength in Cricket Players

**DOI:** 10.7759/cureus.104608

**Published:** 2026-03-03

**Authors:** Shreyash Yedke, Ruchi Kothari, Prashanth A, Manish Rathod, Senthil Kumar

**Affiliations:** 1 Department of Physiology, Mahatma Gandhi Institute of Medical Sciences, Wardha, IND; 2 Department of Physiology, Madha Medical College and Hospital, Chennai, IND

**Keywords:** cricketers, forced vital capacity, grip force transducer, hand grip, pulmonary function

## Abstract

Background

Cricket has emerged as the most popular sport not only in India but also worldwide, with events such as the T20 World Cup, IPL, and Champions Trophy greatly boosting it as a global sport. It is set to feature in the 2028 Los Angeles Olympics. Cricket’s diasporic formats and extremely exhausting play intensities make it a highly physically demanding game requiring exceptional aerobic fitness, muscular endurance, and stamina. Hand grip strength (HGS), an indicator of muscular function, and pulmonary function tests (PFTs), providing qualitative and quantitative evaluation of respiratory efficiency, could prove to be very useful in estimating the overall fitness of a cricketer. As there is limited research on the correlation between pulmonary function and HGS in male cricket players, the present study aimed to explore these parameters and evaluate their relationship.

Methodology

This cross-sectional study was performed over two months in the Sports Physiology Lab of a rural medical college in central India. The study participants were 90 participants, including 30 male cricketers and 60 age-matched controls aged between 17 and 25 years, selected using a convenience sampling technique. Only healthy male cricketers who gave informed consent for participation in the study, and who practised for the last three years, two to three hours daily for six days a week, were included as cases. Pulmonary functions were evaluated using the MIR Spirolab III Diagnostic Spirometer, and HGS was explored using the Grip Force Transducer.

Results

The mean age of the cases was 21.50 ± 1.06 years, and of the controls was 21.31 ± 0.81 years. The difference between study groups with respect to age, height, weight, body mass index (BMI), or blood pressure was not statistically significant. Cricketers showed significantly lower respiratory rate (p < 0.001) and better PFT parameters, with forced vital capacity (FVC), forced expired volume in one second (FEV₁), average forced expiratory flow rate over the middle 50% of the FVC (FEF25-75%), FEV₁/FVC, and peak expiratory flow rate showing better lung function in comparison to controls. Both mean and maximum HGS were also higher in cricketers, with statistical significance. Resting heart rate was lower among cricketers (p = 0.04). Correlation analysis depicted positive but statistically non-significant relationships between pulmonary parameters (FVC, FEF25-75%) and HGS in cricketers.

Conclusions

Cricketers exhibited higher spirometric parameters and better grip strength, reflecting enhanced overall physical fitness in comparison to controls, as demonstrated by a positive association between HGS and PFT indices.

## Introduction

Indian Cricket Team’s victory in major high-profile international events, such as the T20 World Cup, Indian Premier League, Champions Trophy alongwith recent historical win of Indian women cricketers in lifting the ICC World Cup 2025, have sparked a significant rise in interest in this team sport. Cricket has recently been included as an Olympic sport, now ready to mark its dazzling presence in the Los Angeles Olympics 2028.

With the advent of the shorter, more intense formats of the game (such as T20 and one-day cricket), the idea of cricket as a leisurely activity seems ambiguous and makes cricket an undoubtedly challenging and demanding sport [1]. Varied match formats of cricket, differing specialist positions, and the environment in which it is often played require players to face a wide range of playing intensities [2]. The physiological demands of cricket vary depending on the match format and the player’s on-field role, such as bowler or batter. The game is characterized by alternating periods of sustained low-intensity and high-intensity bouts, such as bowling, batting, sprinting between wickets, and fielding [3].

The prerequisites of effective cricket performance include a combination of technical skills, aerobic endurance, and muscular strength. Among all on-field tasks, fast bowling is considered the most physically demanding job, as it requires delivering high-pace balls repetitively, which occurs above 120 times in a day [4]. Fielding is another integral component for both batsmen and bowlers. It calls for considerable aerobic fitness and muscular force for explosive, multidimensional movements.

Christie et al. have documented that there are distinct differences in the physiological and biomechanical profiles; however, bowlers and batters have in common some physical characteristics, such as the demand for rapid sprints while in bowling stances or between wickets [5]. Thus, optimal performance in cricket entails task-specific conditioning of the cricketers. However, there is limited literature on biomechanical analyses of batsmen and bowlers, which is insufficiently researched to date, particularly for male cricketers.

For evaluating and monitoring respiratory health, pulmonary function testing serves as an imperative assessment tool that provides insights into small and large airway functioning, lung parenchymal integrity, and pulmonary capillary functionality [6]. Webster et al. previously reported improvements in respiratory muscle strength and overall pulmonary efficiency with the involvement of individuals in structured physical training in sports such as cricket [7]. In the one-day format, as every inning extends up to 50 overs, bowlers would have to deliver up to 10 overs and may be required to stretch their fielding spell because of the new power-play rules, where there are restricted field placements. These demands impose sustained aerobic activity, muscle strength, and superior respiratory capabilities [7].

Pulmonary function tests (PFTs) provide qualitative and quantitative evaluation of pulmonary function and are crucial in estimating the fitness of a cricketer from a physiological point of view [8]. Spirometry is a frequently employed objective assessment of the respiratory system. In most players, many years of training fall during the growth period, when the lungs are also developing rapidly [9]. Adequate training during that time, keeping targeted pulmonary parameters in mind, may be critical in determining future lung structure and function, including performance of the oxygen transport system [10,11]. In a previous study by Ghosh et al. on sportsmen playing different games, cricket was shown to improve stamina and endurance and demonstrated a significant increase in pulmonary functions [12].

The type and nature of sport have been reported to exert a significant influence on both respiratory function and grip strength [12,13]. The physiological outcome predictor of muscular function assessment is hand grip strength (HGS), which is a rapid, useful, and inexpensive investigation [8]. Grip strength plays a vital role in throwing and casting the ball during bowling and batting across various cricketing disciplines [14]. Among a variety of sports where grasping and force application are important, cricket requires a considerable degree of HGS for optimizing performance and potentially preventing injury [15]. It has been identified as a critical determinant of batting performance. Stretch et al. [16] reported that higher grip strength plays a vital role in enhancing control of the bat during impact of the ball, which improves shot execution and performance. It has also been demonstrated that both respiratory and muscular adaptations are integral in determining athletic performance in a sport like cricket, highlighting the significance of continued research into the physiological correlates of training and on-field play attributes.

Sport-specific variations in training intensity, duration, and physiological demands may differentially affect respiratory muscle performance and hand grip force. Consequently, continued exploration is warranted to investigate the effects of various sports disciplines on these parameters.

Although a few studies [3,17-19] have been reported by previous researchers on isolated measures of HGS and lung function in different populations of players, such as athletes, swimmers, and handball players, substantial evidence regarding the association of pulmonary function and grip strength of cricketers is lacking in the literature.

Hence, the primary aim of the present study was to determine HGS and PFT parameters in male cricket players and to compare them with those of controls. The secondary aim was a correlation analysis between the study variables.

## Materials and methods

Study design and setting

This observational, cross-sectional study was conducted over a duration of two months in the Sports Physiology Laboratory of the Department of Physiology of a rural medical college in central India.

Study population

A total of 30 cricketers with 60 age-matched controls in the age range of 17-25 years were recruited as per the calculated sample size of 90. A convenience sampling technique was utilized for enrolment. OpenEpi 3.01 statistical software (Centers for Disease Control and Prevention, Atlanta) was used to estimate the sample size with the following assumptions: confidence level of 95%, 80% study power, and a case-to-control ratio of 1:2.

Inclusion Criteria for Cases and Controls

Healthy cricketers aged between 17 and 25 years who had played cricket for the last three years and practiced daily for two to three hours for six days a week were included as cases. All participants provided written informed consent. Age-matched individuals not involved in any kind of physical activity who gave consent were recruited as controls.

Exclusion Criteria for Cases and Controls

Individuals suffering from any of the comorbidities, such as chronic respiratory disorders, cardiovascular disorders, systemic diseases, neurological or psychiatric disorders, or musculoskeletal disorders, were excluded from the study.

Ethical approval

Prior approval for the research project was obtained from the Institutional Ethics Committee (approval number: MGIMS/IEC/PHY/91/2023; dated 25/03/2023).

Informed consent

Before conducting the study, participants were briefed about the objective, rationale, requirements, and procedure of the study in detail. After verbal consent was obtained, participants were asked to sign a written consent form as an agreement to be included in this study. Confidentiality was maintained by assigning a unique identification number to each participant. There was no financial burden upon the study participants.

Data sources and measurement of variables

The anthropometric data of both cases and controls were collected. Age was logged in years. The height was measured in bare feet using the Phoenix Height Weight Body Mass Index machine (Model-PBMI 200). The weight of the participants was measured to the nearest 0.1 kg, and then the body mass index (BMI) was displayed on the screen of the same machine.

Grip strength estimation

Grip strength was recorded using Silver-Silver Chloride disposable electrodes placed on the skin surface overlying a muscle of interest. The PowerLab system was used for data acquisition. Analysis was performed by LabChart Pro software from the raw data. Mean and maximum grip strength expressed in Newton (N) were measured for the dominant hand of the participants using the Grip Force Transducer (MLT004/ST) from AD instruments, Australia. The instrument was calibrated in the Sports Physiology Laboratory and checked for adequate quality control before use. Sufficient reference data were established using the same device to ensure its validated usage.

Procedure for Measuring Hand Grip Strength

The dominant forearm of the participant was exposed and thoroughly cleaned using spirit to apply the electrodes. Positive (red) and negative (white) electrodes were placed over the posterior proximal aspect of the forearm on the belly of the muscle, and the ground electrode was applied on the anterior distal aspect of the forearm. The participant was then asked to maintain a standardized arm position while sitting straight without any backrest/support, with the shoulder adducted and neutrally rotated, the elbow flexed at 90°, the forearm in a neutral position, and the wrist in a neutral position. Two trials at intervals of 20 seconds were given to the participants to record the maximum value of the grip strength. The best of the two values was taken as the maximum, which corresponded to 100% of the maximum grip strength. Participants were asked to apply 30% of their strength on the Grip Force transducer in their dominant hand for about one to three minutes. Preliminary trials were given on the non-dominant hand to enable the participant to familiarize themselves with the system for maintaining 30% of the grip [20].

Pulmonary function tests

Pulmonary functions were studied by spirometry using a computerized spirometer (MIR Spirolab III Diagnostic Spirometer, Ahmedabad, Gujarat, India), adhering to the guidelines of the American Thoracic Society and European Respiratory Society [21]. The device was duly calibrated by standardized procedures beforehand. The following parameters were evaluated: (1) forced vital capacity (FVC), which is the maximum volume of air that can be exhaled forcefully after a maximal inspiration. (2) Forced expired volume in one second (FEV₁), which is the volume expired in the first second of maximal expiration following a maximal inspiration. (3) FEV₁/FVC: FEV₁, i.e., percentage of the FVC, is a valuable indicator of airflow obstruction. (4) Peak expiratory flow rate (PEFR), which is the highest flow (measured in L/minute or L/second) achieved during maximally forced expiration following a full inspiration. (5) Average forced expiratory flow rate over the middle 50% of the FVC (FEF25-75%), which is known as the maximum flow rate during 25-75% of the expired volume or the maximum mid-expiratory flow rate. The average value ranges between 25% and 75% of the FVC, measured in L/second. It is considered a more sensitive index of small airway function. (6) Maximum voluntary ventilation (MVV), which is the volume of air that can be expelled and inhaled in one minute. It is useful for assessing respiratory muscle strength.

Statistical analysis

Statistical analysis was performed employing an unpaired Student’s t-test for continuous variables. The mean and standard deviation of all studied continuous variables in normal distribution were assessed using SPSS version 20.0 (IBM Corp., Armonk, NY, USA). Linear regression analyses were performed using standard techniques. Pearson’s correlation test was used to examine the correlation between variables. The correlation coefficient (r) was analyzed for statistical significance. A p-value <0.05 was kept as the level for statistical significance.

## Results

This study included 30 cricketers in the age range of 17-25 years and 60 age-matched healthy individuals as controls. The mean age of the cases was 21.50 ± 1.06 years, and of the controls was 21.31 ± 0.81 years. The descriptive statistics of the two study groups are presented in Table 1. The difference between the means of age, height, weight, BMI, and systolic and diastolic blood pressure was not statistically significant (p > 0.05).

**Table 1:** Descriptive statistics of the study groups *: All reported p-values are based on Student’s t-test between the case and control groups. SD: standard deviation; BMI: body mass index; BP: blood pressure; NS: non-significant; HS: highly significant

Parameters	Cases (n = 30), mean ± SD	Controls (n = 60), mean ± SD	P-value*
Age (years)	21.50 ± 1.06	21.31 ± 1.81	0.592 (NS)
Height (cm)	173.5 ± 5.68	171.69 ± 5.27	0.138 (NS)
Weight (kg)	70.50 ± 11.94	67.83 ± 11.63	0.311(NS)
BMI (kg/m²)	23.43 ± 4.25	23.00 ± 3.84	0.630 (NS)
Respiratory rate (breaths/minute)	14.76 ± 1.55	16.18 ± 1.10	<0.0001(HS)
Systolic BP (mmHg)	119.76 ± 7.81	120.66 ± 8.45	0.70 (NS)
Diastolic BP (mmHg)	79.94 ± 8.44	77.78 ± 7.23	0.31 (NS)

As shown in Table 2, in relation to the control group, cases had significantly higher values of all the PFT parameters.

**Table 2:** Pulmonary function test parameters of the study groups. *: All reported p-values are based on Student’s t-test between the case and control groups. FVC: forced vital capacity; FEV₁: forced expired volume in one second; PEFR: peak expiratory flow rate; FEF25-75%: average forced expiratory flow rate over the middle 50% of the FVC; MVV: maximum voluntary ventilation; S: significant; HS: highly significant

Parameters	Cases (n = 30), mean ± SD	Controls (n = 60), mean ± SD	P-value*
FVC (L)	4.5 ± 0.54	3.26 ± 0.60	<0.0001 (HS)
FEV₁ (L)	4.19 ± 0.46	2.90 ± 0.63	<0.0001 (HS)
FEF25-75% (L/second)	5.09 ± 1.28	4.25 ± 0.93	0.0006 (S)
FEV₁/FVC (%)	93.50 ± 5.64	89.33 ± 8.24	0.014 (S)
PEFR (L/second)	9.41 ± 1.85	6.51 ± 1.89	<0.0001 (HS)
MVV (L/minute)	131.38 ± 21.45	112.09 ± 17.27	<0.0001 (HS)

The HGS indices of males in both the study groups were expressed as mean ± standard deviation (Table 3). The differences in the HGS indices, namely, mean HGS and maximum HGS, when compared between the two groups, were statistically significant. A statistically significant difference was found between the heart rate of the two study groups (p = 0.04).

**Table 3:** HGS indices of the study groups. *: All reported p-values are based on Student’s t-test between the case and control groups. HGS: hand grip strength; SD: standard deviation; S: significant; HS: highly significant

Parameters	Cases (n = 30), mean ± SD	Controls (n = 60), mean ± SD	P-value*
Resting heart rate (beats/minute)	74.18 ± 8.09	80.06 ± 10.87	0.04 (S)
Max grip strength (N)	451.50 ± 78.81	334.52 ± 62.67	<0.0001 (HS)
Mean grip strength (N)	100.5 ± 17.31	90.69 ± 13.40	0.003 (S)

**Figure 1:**
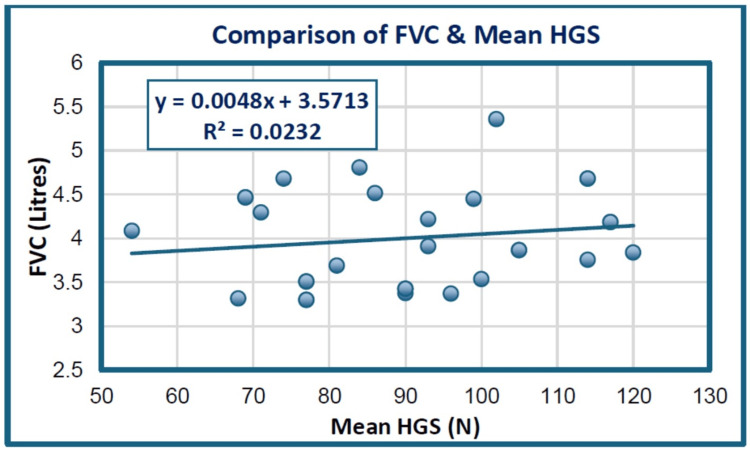
Scatterogram showing the correlation between FVC and mean HGS among the study groups. HGS: hand grip strength; FVC: forced vital capacity

As shown in Table 4 and the scatterogram in Figure 1, showing the analysis between FVC and mean HGS, there was a statistically non-significant positive correlation (r = 0.152, p = 0.406), indicating an increase in FVC with a corresponding increase in the mean HGS value.

**Table 4:** Correlation of FVC with mean HGS between case and control groups. HGS: hand grip strength; FVC: forced vital capacity; r: Pearson’s correlation coefficient

Study group	FVC (L), mean ± SD	HGS (N), mean ± SD	r value	P-value
Male cricketers	4.5 ± 0.54	100.5 ± 17.31	0.152	0.406
Control males	3.26 ± 0.60	90.69 ± 13.40	-0.03	0.843

**Figure 2:**
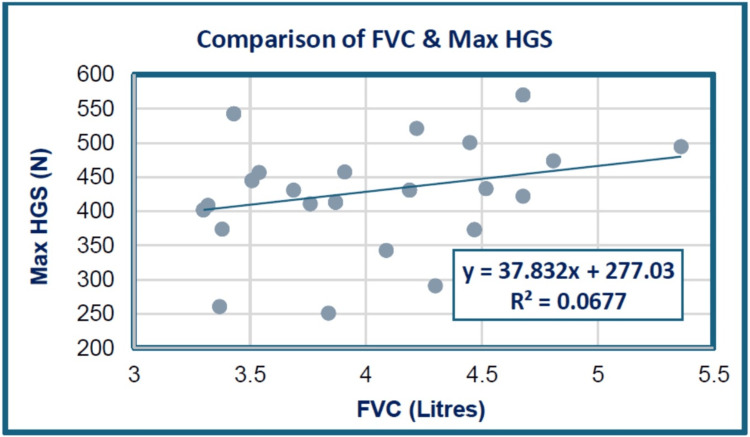
Scattergram showing the correlation between FVC and maximum HGS among the study groups. HGS: hand grip strength; FVC: forced vital capacity

As shown in Figure 2 and Table 5, between FVC and maximum HGS in male cricketers, a positive correlation (r = 0.260, p = 0.147) was achieved, which was statistically non-significant.

**Table 5:** Correlation of FVC with maximum HGS between the case and control groups. HGS: hand grip strength; FVC: forced vital capacity; r: Pearson’s correlation coefficient

Study group	FVC	Maximum HGS (N)	r value	P-value
Male cricketers	4.5 ± 0.54	451.50 ± 78.81	0.260	0.147
Control males	3.26 ± 0.60	334.52 ± 62.67	0.233	0.242

**Figure 3:**
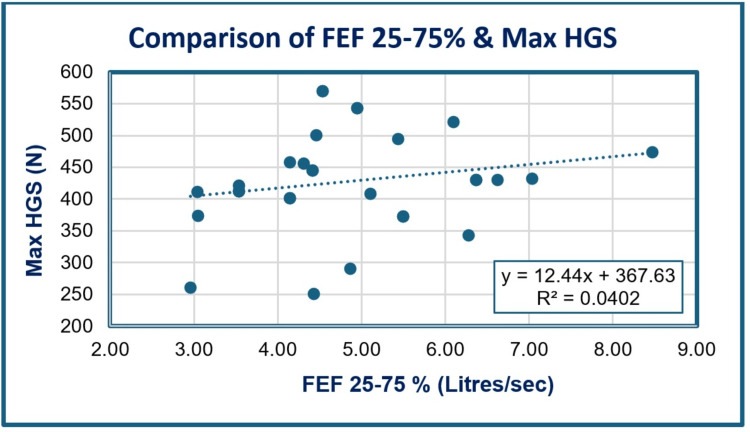
Scattergram showing the correlation between FEF25-75% and maximum HGS among the study groups. HGS: hand grip strength; FEF25-75%: average forced expiratory flow rate over the middle 50% of the forced vital capacity

As depicted in Table 6 and Figure 3, a positive correlation (r = 0.200, p = 0.272) was obtained between FEF25-75% and maximum HGS of male cricketers, which was statistically non-significant.

**Table 6:** Correlation of FEF25-75% with maximum HGS between the case and control groups. HGS: hand grip strength; FEF25-75%: average forced expiratory flow rate over the middle 50% of the forced vital capacity; r: Pearson’s correlation coefficient

Study group	FEF25-75% (L/second)	Maximum HGS (N)	r value	P-value
Male cricketers	5.09 ± 1.28	451.50 ± 78.81	0.200	0.272
Control males	4.25 ± 0.93	334.52 ± 62.67	-0.01	0.960

**Figure 4:**
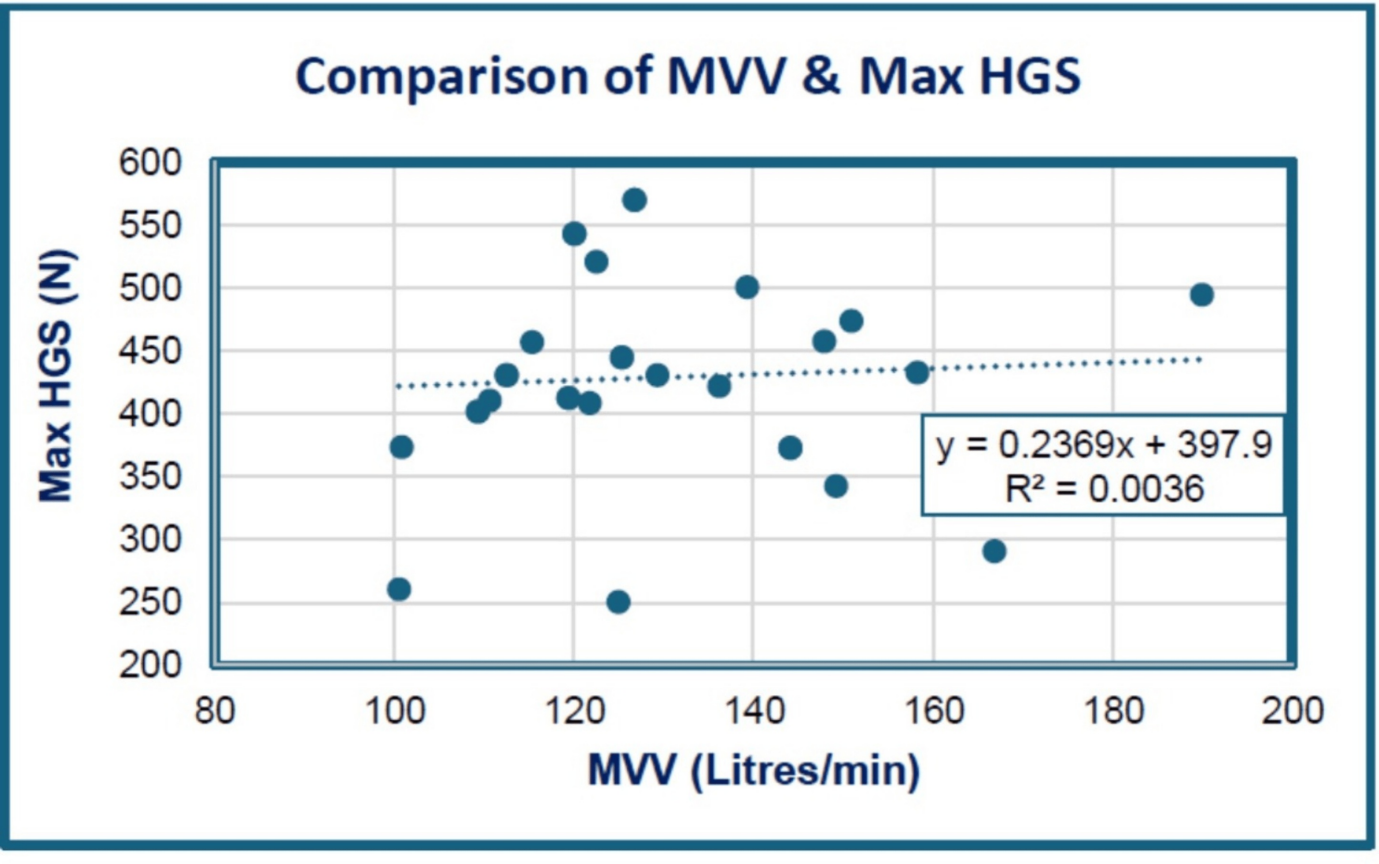
Scattergram showing the correlation between MVV and maximum HGS among the study groups. HGS: hand grip strength; MVV: maximum voluntary ventilation

As illustrated in Figure 4 and Table 7, between MVV and maximum HGS in male cricketers, a positive correlation (r = 0.05, p = 0.785) was achieved, which was statistically non-significant.

**Table 7:** Correlation of MVV with maximum HGS between the case and control groups. HGS: hand grip strength; MVV: maximum voluntary ventilation; r: Pearson’s correlation coefficient

Study group	MVV (L/minute)	Maximum HGS (N)	r value	P-value
Male cricketers	131.70 ± 19.82	451.50 ± 78.81	0.05	0.785
Control males	112.09 ± 17.27	334.52 ± 62.67	0.372	0.05

## Discussion

Although it is known that developing grip strength and lung function can benefit performance in a game like cricket, there is scant research on the physiological and physical correlates of cricketers. Though the connection between HGS and PFTs has been explored in earlier studies [4,10,11,14,15], only a few have reported how this relationship varies in cricketers. Due to the limited literature on men’s cricket, this study primarily focused on male cricketers and conducted an investigation of batters and bowlers of different skill sets. The present study was undertaken to evaluate associations between indices of HGS and PFT parameters in male cricketers.

The available reported pulmonary function values of cricketers studied abroad [10,11] were higher than those of their counterparts studied in our region. Such variations might be due to the ethnicity and differences in age, physique, and aerobic fitness scores, which alter pulmonary parameters.

In the study by Durmic et al. [11] on 150 male athletes from four different sports, the PEFR (9.4 ± 2.3 L/second) obtained in the soccer players was similar to that noted in the cricketers included in our study at 9.41 ± 1.85 L/second, whereas their FVC and MVV were higher than those of the present study. Another parameter, i.e., FEV₁/FVC (93.50 ± 5.64) in our study, was found to be higher than theirs (84.6 ± 7.2).

Pulmonary function parameters of trained Indian sportsmen belonging to different sports activities and of 10 sedentary individuals were studied by Ghosh et al. [12]. According to their study, the mean MVV and FEV₁ in all sportspersons (athletes, badminton, basketball, swimming, volleyball, hockey, and table tennis players) were better than those of our sedentary controls, which is in line with the results of our study. The mean FEV₁ of almost all the groups of sportsmen exhibited higher values than those reported by other studies conducted among sedentary individuals in India [1,12].

Every sport is different regarding the kind and intensity of exercise that is performed, varying with the season, and there are sport-specific alterations in body composition, which has been coined as “sport-specific morphological optimization” [22,23].

Asha et al. [14] found that the mean HGS was significantly higher in cricket batsmen than in the controls, which is consistent with our observations. The highest hand grip scores were recorded in the dominant hand of six of eight right-handed batsmen. It is suggested that possessing higher HGS positively influences batting performance, particularly when controlling the bat during ball impact.

Alajam et al. [24] conducted a study among 50 healthy young male college students in Nigeria and recorded PFTs using a spirometer with HGS through an electronic handheld dynamometer. The mean age of their participants was 21.72 ± 1.49 years, which is close to that of our study (21.31 ± 1.81 years); hence, their results were comparable to ours. Pearson’s correlation analysis in their study showed that the dominant HGS was significantly positively correlated with FVC (r = 0.55, p < 0.001) and FEV₁ (r = 0.56, p < 0.001).

Similarly, in our study, there was a positive correlation of FVC with mean HGS (r = 0.152) and FVC with maximum HGS (r = 0.260). These results suggest that regular physical activity and training sessions might have led to better pulmonary function and HGS. This observation of better HGS is in concordance with Asha et al. [14], who conducted a cross-sectional analytical study at Dhaka Medical College, Dhaka, Bangladesh, among 50 Bangladeshi male sprinters and 50 Bangladeshi male cricket batsmen.

According to Akınoğlu et al. [25], the underlying link between HGS and pulmonary function may be due to common physiological pathways integrating the muscular and respiratory systems. Peripheral muscles play an important role in effective rhythmic breathing, and their strength can positively influence respiratory capacity. Training sessions of cricketers ultimately aim at strengthening peripheral muscles, which would result in better pulmonary parameters and improved respiratory muscle strength.

Follow-up longitudinal studies [25,26] have pointed toward the conclusion that higher HGS tends to maintain better respiratory indices over time, which suggests that maintaining or augmenting peripheral muscle strength results in increasing respiratory efficiency. These findings are in line with previous research [10,11] emphasizing the role of muscular strength in respiratory health and highlighting that exercises focused on building peripheral muscle strength would optimize lung efficiency.

Study limitations

The study has a few limitations to be acknowledged. First, its cross-sectional study design limited the ability to decipher a causal relationship between HGS and PFT parameters. Second, the participant pool comprised only males aged 17-25 years, which limits the generalizability of the findings to other age groups or females. Furthermore, as all participants were recruited from a single setting, the results may not be directly generalizable to young adult males in other regions with differing demographic or health profiles. The small number of participants in each study group may not reflect the true scenario of the cricketing population. As a convenience sampling technique was employed, it would have limited the representativeness of the study population. Participants were recruited based on accessibility rather than random selection, thereby restricting the extrapolation of the findings to the broader target population. The absence of probability-based sampling reduces external validity and could be a cause of sampling bias. The adjustment for potential confounders was not comprehensively performed. Some correlations observed in the study did not reach statistical significance, due to which potential, genuine associations might have been missed. Hence, the results should be interpreted with caution, and further studies with larger sample sizes are required to potentiate the findings.

Study strengths

Despite some shortcomings, our study possesses certain strengths. To our knowledge, this is probably among the preliminary studies that have examined the correlation between HGS and pulmonary function parameters in young male cricketers of this region while controlling for potential confounding factors. Grip Force transducer, a sophisticated device, was employed to assess HGS in the study subjects, which made the investigation more efficacious, easy to analyze, simple, and less time-consuming at the same time.

Future directions

Clearly, there is a need for longitudinal research targeting both men and women cricketers, exploring the relationship between handgrip and pulmonary function spanning over time. It would eventually help assess how alterations in muscle strength impact the aerobic fitness and overall health outcomes.

## Conclusions

The results of this study highlight a positive association of HGS with pulmonary function indices among young male cricketers who had better respiratory efficiency, parallelled with greater peripheral muscle strength in comparison to controls. This composite battery of tests may serve as a practical tool for evaluating the overall muscular strength and respiratory function of cricket players.
